# Cerebellar large B-cell lymphoma: a case report

**DOI:** 10.1186/s13256-018-1880-z

**Published:** 2018-11-17

**Authors:** Malik Ghannam, Shaden Mansour, Fareed Jumah, Brent Berry, Albertine Beard

**Affiliations:** 1 Department of Neurology, University of Minnesota, Minneapolis, MN USA; 2 An-Najah National University, Nablus, West Bank Palestine; 3 Minneapolis Veterans Affairs Healthcare System, Section of Hospital Medicine, Minneapolis, MN USA; 4 Department of Internal Medicine, University of Minnesota School of Medicine, Minneapolis, MN USA

**Keywords:** Literature review, Case report, Primary central nervous system lymphoma, Large B-cell lymphoma, Cerebellum, Immunocompetent, Posterior circulation stroke, Rare condition

## Abstract

**Background:**

Primary central nervous system lymphoma is a rare, malignant non-Hodgkin lymphoma that can arise in the brain, spinal cord, eye, leptomeninges, or cranial nerves. Primary central nervous system lymphoma is rare, accounting for 2–6% of all primary brain neoplasms and 1–2% of all non-Hodgkin lymphomas, and it usually presents as a solitary lesion. Cerebellar involvement is present in only 9% of cases. We present an unusual case of primary central nervous system lymphoma presenting as multiple lesions in the cerebellum in an immunocompetent host.

**Case presentation:**

A 71-year-old Caucasian man presented to our hospital with acute onset of dizziness, nausea, vomiting, and gait imbalance. Contrast-enhanced computed tomography revealed three intensely enhancing masses in the right cerebellar hemisphere. Whole-body positron emission tomography and computed tomography failed to demonstrate a primary tumor of origin outside the central nervous system. The patient underwent right suboccipital craniotomy with partial resection of the visible tumor from the right cerebellum. Histopathology revealed diffuse large B-cell lymphoma, non-germinal center type.

**Conclusions:**

Primary central nervous system lymphoma is rare, even more so in the cerebellum. However, the overall incidence of primary central nervous system lymphoma is rising in both immunocompromised and immunocompetent patients. The highly aggressive nature of primary central nervous system lymphoma necessitates timely diagnosis and intervention. In this report, we review the available literature for a better understanding of the pathophysiology and management of primary central nervous system lymphoma. To the best of our knowledge, this is the first reported case of a patient with primary central nervous system lymphoma presenting with multiple masses in the cerebellum.

## Background

Primary central nervous system lymphoma (PCNSL) is a rare, malignant non-Hodgkin lymphoma (NHL) that is confined to the central nervous system. Most cases are diffuse large B-cell lymphoma. PCNSL is an aggressive tumor that extensively invades the parenchyma but by definition remains confined within the central nervous system (stage IE) [1, 2]. PCNSL is rare, accounting for only 2–6% of all primary brain neoplasms and 1–2% of all NHLs [2]. However, in the last couple of decades, its incidence has been rising among the immunocompetent elderly population, but not in younger individuals. Survival remains poor, regardless of the patient’s immune status [3].

PCNSL presents as a solitary lesion in 60–70% of patients, most commonly in the hemispheres (38%), thalami/basal ganglia (16%), corpus callosum (14%), periventricular regions (12%), and rarely in the cerebellum (9%) [4]. We present an unusual case of PCNSL presenting as multiple lesions in the cerebellum in an immunocompetent host.

## Case presentation

A 71-year-old Caucasian man presented to the emergency department of our hospital with a 1-week history of abrupt-onset blurry vision, dizziness, nausea, vomiting, and ataxia initially thought consistent with a posterior circulation stroke. The patient denied associated vertigo or headache. He had no prior history of stroke and had been taking prophylactic aspirin for years for a patent foramen ovale. Noncontrast head computed tomography (CT) performed in the emergency department demonstrated no visible masses or hemorrhage. A shrapnel adjacent to the patient’s eyes precluded the possibility of further visualization with magnetic resonance imaging (MRI). He was admitted for further workup and treatment. Carotid Doppler ultrasound showed no stenosis. Subsequent CT angiography did not clearly visualize the brain parenchyma but showed no vascular compromise. The initial working diagnosis was of a cerebellar stroke, and the patient was transferred to the acute inpatient stroke rehabilitation service.

**Fig. 1:**
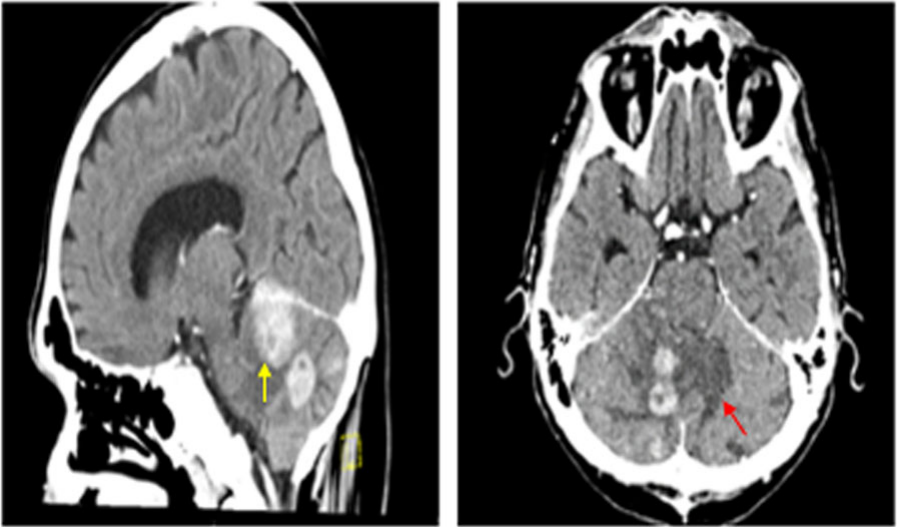
CT with contrast showing enhancing cerebellar lesion (yellow arrow) and vasogenic edema (red arrow)

**Fig. 2:**
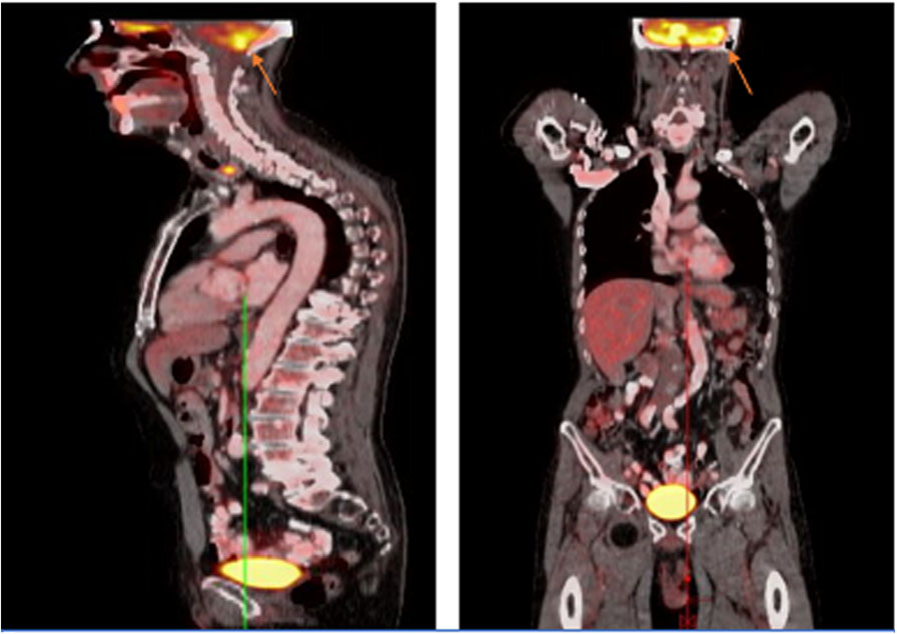
PET scan showing cerebellar hypermetabolic lesions (orange arrow)

**Fig. 3:**
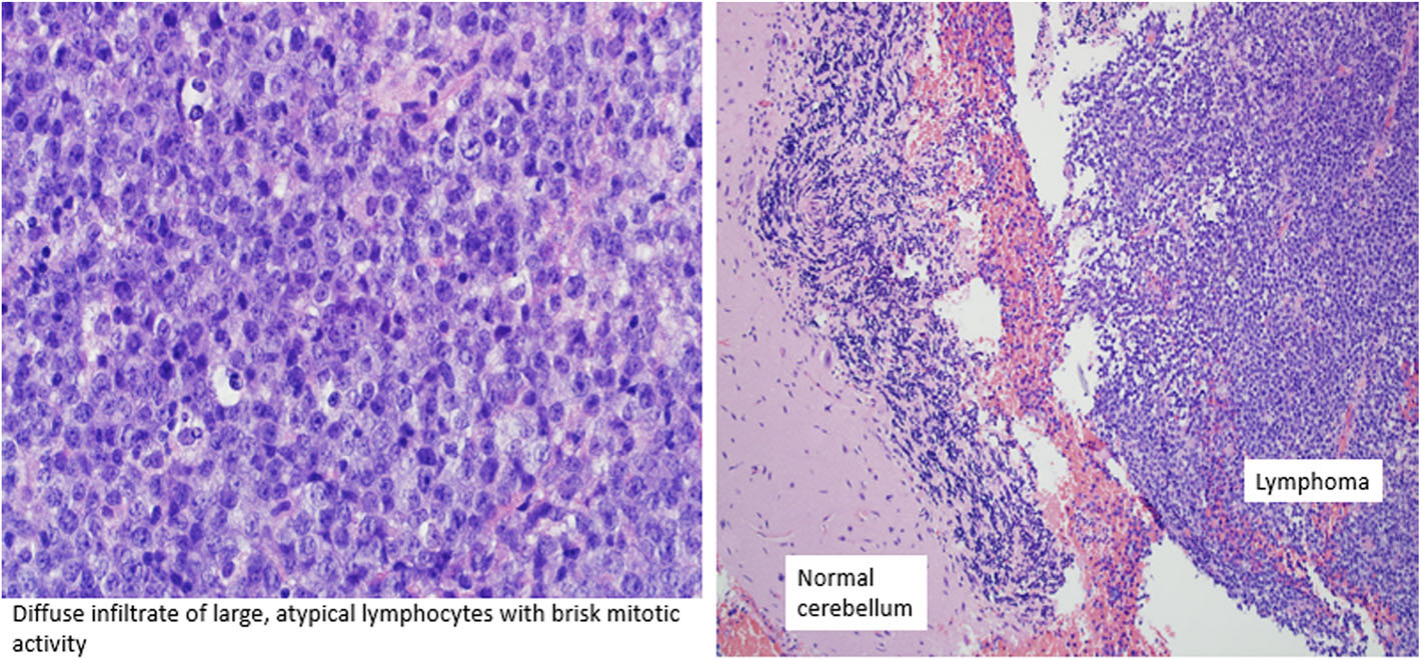
Histopathological slide showing diffuse infiltrate of large, atypical lymphocytes with brisk mitotic activity of the cerebellar lesion

**Fig. 4:**
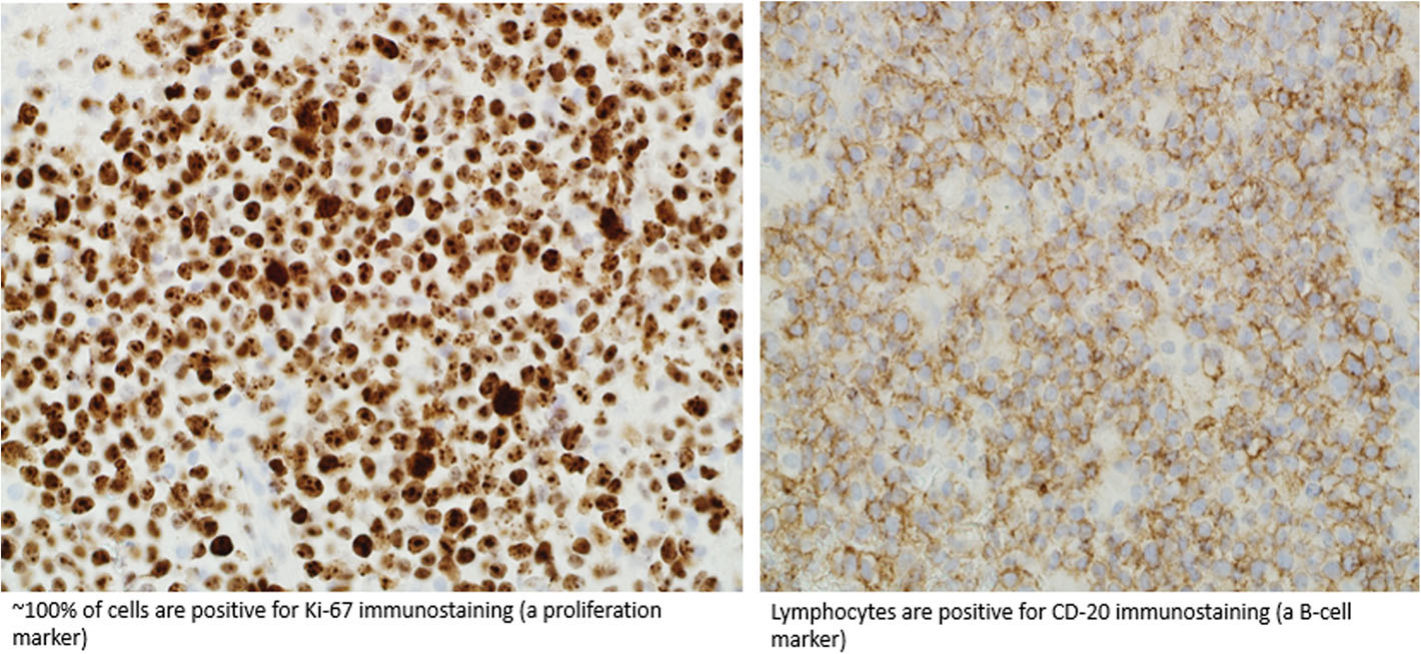
Slide showing that ~ 100% of cells are positive for Ki-67 immunostaining (a proliferation marker) and that lymphocytes are positive for CD-20 immunostaining (a B-cell marker) of the cerebellar lesion

Despite participation in rehabilitation therapies, his symptoms progressively worsened, prompting repeat noncontrast head CT 9 days after admission, which demonstrated indistinct, masslike lesions in the cerebellum, one with evidence of hemorrhage and surrounding vasogenic edema and mild hydrocephalus. Contrast-enhanced CT performed later that day revealed three intensely enhancing masses in the right cerebellar hemisphere (Fig. 1). The patient was started empirically on steroids for his vasogenic edema, which produced rapid improvement in his symptoms. Because these cerebellar lesions appeared most consistent with metastatic disease, the neurosurgery service recommended metastatic cancer workup without immediate surgical intervention. CT with contrast enhancement and whole-body positron emission tomography failed to demonstrate a primary tumor of origin outside the central nervous system (Fig. 2). The patient underwent right suboccipital craniotomy with partial resection of the visible tumor in the right cerebellum. Histopathology revealed diffuse large B-cell lymphoma, non-germinal center type (Figs. 3 and 4).

Bone marrow biopsy and testicular ultrasound demonstrated no evidence of lymphoma in these sites. The result of human immunodeficiency virus (HIV) testing was negative. The patient elected to pursue induction chemotherapy. Because of his age, the radiation oncology service recommended against whole-body irradiation to minimize neurotoxicity. The patient was started on the MATRix regimen (methotrexate, cytarabine, and rituximab), which the patient has tolerated well thus far, with no residual disease seen on contrast-enhanced head CT scans. The patient has experienced marked improvement in his symptoms with treatment and is able to ambulate with a walker, but he still reports balance problems and blurry vision.

## Discussion

PCNSL is a malignant NHL. It was first described by Bailey in 1929 as perivascular sarcoma [5]. The vast majority (90%) of cases are diffuse large B-cell lymphomas. Less common variants include Burkitt, T-cell, immunoblastic, or low-grade malignant B-cell lymphomas [1, 5, 6]. PCNSL can arise in the brain, spinal cord, eyes, cranial nerves, or meninges [7]. The aggressive parenchymal involvement of PCNSL almost always invades only locally, rarely metastasizing outside the nervous system. PCNSL itself is rare, comprising only 2–6% of all primary brain tumors and 1–2% of all NHLs [2]. However, its incidence is rising the fastest among all intracranial tumors [8]. The median age at diagnosis is 53–57 years in immunocompetent patients, as in our case, the patient was immunocompetent when he was diagnosed with a 1.2:1 male-to-female sex distribution, whereas in immunocompromised patients, the median age at diagnosis is 31–35 years with a clear male predominance (male-to-female ratio of 7.38:1) [9]. Immunocompromise in these patients is typically secondary to HIV, organ transplant, or a primary immunodeficiency syndrome [10] such as ataxia telangiectasia; severe combined and common variable immunodeficiency; Wiskott-Aldrich syndrome; or autoimmune diseases such as rheumatoid arthritis, systemic lupus erythematosus, myasthenia gravis, and sarcoidosis [9].

The location of the PCNSL determines the clinical picture. The most common presentation, seen in 70% of patients, is focal neurological symptoms. Forty percent present with neuropsychiatric symptoms, followed in frequency by signs of increased intracranial pressure such as headache, nausea, and vomiting in 33%, seizure in 14%, and ocular symptoms in 4% of cases [11]. Determining a diagnosis of PCNSL can be challenging, owing to the absence of stereotypical clinical symptoms, the heterogeneity of the pathological morphology, the lack of specific diagnostic laboratory testing, and the variable appearance on imaging [12]. Initial evaluation should include head CT and MRI to visually characterize the lesion; scanning of the chest, abdomen, and pelvis and testicular ultrasound, bone marrow biopsy, and slit-lamp examination to evaluate for other sites of lymphoma involvement; and HIV testing [13]. Imaging-guided stereotactic biopsy, including histopathology and immunohistochemical staining, remains the preferred method of diagnosing PCNSL [10]. Macroscopically, PCNSL appears as a well-circumscribed, firm, gray to tan-yellowish mass with areas of hemorrhage [9]. PCNSL has a characteristic appearance on both CT and MRI scans [14], commonly appearing isodense or hyperdense on CT scans and enhancing with contrast [15]. Microscopically, it shows a characteristic angiocentric growth pattern with splitting of blood vessels [13]. PCNSLs are typically derived from late germinal center exit B cells [16]. Thus, tumor cells often express pan B-cell markers (cluster of differentiation 19 [CD19], CD29, CD79a) and in 90% of cases express BCL-6 and MUM1, whereas plasma cell-associated proteins such as CD38 and CD138 are usually absent [17].

PCNSL usually presents as a single homogeneous mass with surrounding edema; therefore, a wide differential diagnosis must be considered because many malignant and benign lesions present with a similar radiographic pattern. Malignant lesions that can appear similar include glioblastoma multiforme (GBM) and brain metastasis from primary cancers such as prostate and lung adenocarcinomas. Although histologically high-grade gliomas such as GBM have a greater degree of cellular and nuclear pleomorphism, infiltrative borders, and central necrosis with pseudopalisading, radiologically they can be difficult to distinguish from PCNSL because both can cross the corpus callosum (“butterfly pattern”) [18] and can present with subependymal lesions [19]. However, PCNSL exhibits more restricted diffusion and lower apparent diffusion coefficient values on diffusion-weighted imaging (DWI) than GBM [2]. PCNSLs are hypointense on T2-weighted MRI signals owing to low intratumoral water content, unlike gliomas, metastasis, and tumefactive demyelinating lesions, which are T2-hyperintense [18, 20]. However, brain metastases are more likely than PCNSL to have blood products, and they present as multiple enhancing lesions at the gray-white matter junction (from hematogenous spread) [19], though our patient’s case highlights that these distinctions are not always diagnostically useful.

Benign lesions with radiographic appearance similar to PCNSL include subacute infarction, demyelinating diseases, infectious lesions, and neurosarcoidosis. Rich macrophage infiltrates seen on biopsy may point toward infarction or demyelination. Demyelinating disorders are characterized by axonal sparing, whereas infarcts show axonal loss. One particularly important infectious etiology to consider is toxoplasmosis, which is the most common brain lesion in patients with HIV. Distinguishing features include greater diffusion on DWI and clinical improvement with pyrimethamine-sulfadiazine can help establish a diagnosis of toxoplasmosis [21]. Neurosarcoidosis rarely presents as a tumor-like lesion within the brain, but on MRI it displays T2-hypointensity with perivascular dissemination and variable contrast enhancement without necrosis.

The clinical picture of PCSNL can also mimic posterior circulation stroke presentation, as did our patient. Therefore, this condition must be excluded, keeping in mind that it represents nearly 20% of all ischemic strokes. A posterior circulation stroke is defined as infarction of the vertebrobasilar arterial system [22]. The underlying pathophysiology involves occlusion or embolism (from large vertebrobasilar atherosclerosis or from the heart), dissection, vasculitis, and dolichoectasia (elongation and tortuosity of the vertebral and basilar arteries), among other less common causes. The posterior circulation mainly supplies the cerebellum, brainstem, and occipital cortex, and symptoms of stroke usually range from dizziness (47%) to unilateral limb weakness and dysarthria (41% and 31%, respectively). Posterior circulation stroke should be suspected in patients presenting with acute neurological symptoms, and this is diagnosed on the basis of history and clinical examination mainly, assisted by imaging studies. Diffusion-weighted MRI is the imaging modality of choice. Acute-phase CT provides suboptimal visualization of the posterior fossa structures; however, it allows identification of large-vessel occlusions or dissection if MRI is contraindicated or unavailable [23]. Subtle hypodensities, sulcal effacement, and loss of gray-white matter differentiation have been used to assess for signs of early ischemia on noncontrast head CT. Current international guidelines recommend conservative management, including the use of antiplatelet agents or anticoagulants. However, these interventions can be complicated with intracerebral hemorrhage or the need of ventriculoperitoneal shunting for hydrocephalus. Microsurgical intervention for cranial nerve compression may be lifesaving, and superficial temporal artery and superior cerebellar artery bypass have been reported [22].

Many conditions can mimic posterior circulation ischemic stroke. Acute peripheral vestibular dysfunction is much more common and typically causes vertigo only with no other neurological symptoms. A head impulse test or Dix-Hallpike maneuver may aid in diagnosis. Acute intracranial hemorrhage, subarachnoid hemorrhage, and tumors may also mimic posterior stroke, which can be distinguished using proper imaging. Moreover, basilar migraine with aura should always be excluded, as well as other metabolic disturbances, such as hypoglycemia or central pontine myelinolysis, which can initially present with stroke-like features. Sarcoidosis or Whipple and Behçet disease can present acutely but with preceding systemic clinical features. Antibody-associated disorders such as Miller Fisher syndrome (ophthalmoplegia, ataxia, and areflexia) should be excluded as well [23].

Unfortunately, PCNSL is an aggressive tumor with high rates of recurrence after treatment. It has a poor prognosis without treatment, with an expected survival of only 3–6 months. Chemotherapy alone or combined with radiation can boost the estimated survival time up to 25–60 months [24]. Relapses occur in the first and second years in 30–60% of patients, with survival after relapse averaging 2–4 months [25].

The combination of chemotherapy and radiation is the standard treatment and can markedly improve the poor survival rates. High-dose methotrexate combined with whole-brain radiation has been widely used [26]. The preferred radiation therapy includes whole-brain radiation of 40–50 Gy followed by local radiation on regions of edema of 60 Gy. Other combination chemotherapies may be used, including methotrexate, temozolomide, and rituximab [27]. Rarely is surgical excision of these tumors possible, owing to their typically deep location, carrying significant risk of postoperative neurological complications. Moreover, resection carries no benefit in survival rates. Surgical resection should routinely be used only in cases of solitary lesions and in cases where intracranial pressure is increased [28].

## Conclusions

PCNSL is a rare disease with an increasing incidence in both immunocompromised and immunocompetent individuals. Our patient, who had multiple lesions in the cerebellum, highlights two unusual presentations of this rare disease that made the initial diagnosis particularly challenging. Because imaging in this disease is nondiagnostic, biopsy is crucial to differentiating PCNSL from malignant and benign lesions that present in a similar manner and appear similar on imaging. The highly aggressive nature of PCNSL necessitates timely diagnosis and intervention. Therefore, physicians must include this disease in their differential diagnosis of mass lesions found in the central nervous system.

## Abbreviations

CD: Cluster of differentiation
DWI: Diffusion-weighted imaging
GBM: Glioblastoma multiforme
HIV: Human immunodeficiency virus
MRI: Magnetic resonance imaging
NHL: Non-Hodgkin lymphoma
PCNSL: Primary central nervous system lymphoma
